# A unified qualitative-quantitative method to evaluate the impact of being a near-peer health educator

**DOI:** 10.1093/eurpub/ckac131.369

**Published:** 2022-10-25

**Authors:** D Arva, A Cseh, Á Mészáros, D Major, A Jeney, D Dunai, S Zörgő

**Affiliations:** 1 Department of Public Health, Semmelweis University, Faculty of Medicine, Budapest, Hungary; 2 Baltic-Black Sea Regional Studies Programme, Ivan Franko National University of Lviv, Faculty of International Relations, Lviv, Ukraine; 3 Interdisciplinary Social Research PhD Program, Eötvös Loránd University, Faculty of Social Sciences, Budapest, Hungary; 4 Care and Public Health Research Institute, Maastricht University, Faculty of Health, Medicine and Life Sciences, Maastricht, Netherlands

## Abstract

Grasping the complexity of public health interventions is of increasing interest. Program evaluation may involve previously known and unknown variables; the former are best explored with quantitative, the latter with qualitative methods. As part of the impact evaluation of the Balassagyarmat Health Education Program (BEP), a near-peer education intervention targeting adolescents from a disadvantaged region of Hungary, we aimed to understand the complex effects of being an educator on medical students’ knowledge about the biopsychosocial model of health. Thus, we developed a unified method that enables us to conduct an exploratory study on the effects of our intervention, then quantify and model that qualitative data. We started the method design with literature review and consultations with methodological and public health experts. We then refined the research questions based on a focus-group discussion held with 6 peer educators. After a set of pilot-interviews, we chose simulation interviewing as our knowledge elicitation procedure, then finalized the protocol with the help of additional piloting. In this unified method, simulation interviews are administered to peer educators and aligned controls, and cognitive task analysis is performed with the help of visual stimuli. Codes are developed inductively and, along with segmentation procedures, applied deductively to the entire dataset via the Reproducible Open Coding Kit. Resulting quantified narratives are further processed with Epistemic Network Analysis. The relative frequency of code co-occurrence in each segment is modelled with networks enabling the qualitative and statistical comparison of data between subsamples. Building on the benefits of qualitative and quantitative approaches, this method offers a complex evaluation of the impact of health education interventions. By strengthening the methods of program evaluation we aim to facilitate the development of more effective interventions.

**Key messages:**

• Qualitative and quantitative methods can be unified in program evaluation to promote a deeper understanding of the complexity in public health interventions.

• Cognitive task analysis as a knowledge elicitation procedure can be used for the impact evaluation of health education programs.

